# Public Health Research and Program Strategies for Diabetes Prevention and Management

**DOI:** 10.5888/pcd22.240501

**Published:** 2025-03-20

**Authors:** Shelly-Ann Bowen, Arsham Alamian, Stephen Onufrak

**Affiliations:** 1 Centers for Disease Control and Prevention, Division of Diabetes Translation, Atlanta, Georgia; 2 University of Miami, Coral Gables Campus, School of Nursing and Health Studies, Coral Gables, Florida

The year 2025 commemorates 50 years since Congress received the Report of the National Commission on Diabetes that established “the urgent need to address directly and fully the tragedy of diabetes mellitus” (1). The 1975 report indicated that the prevalence of diabetes had increased by 50% over the preceding decade, resulting in the condition affecting 5% of the population at that time. Since then, largely because of substantial increases in obesity, the prevalence of diabetes in the US has more than doubled, now nearing 12% of people in the US (2). Furthermore, notable disparities persist in the prevalence of type 2 diabetes, observed across characteristics such as race and ethnicity, socioeconomic status, and whether individuals live in rural or urban areas (2). Despite these challenges, in the past 50 years, public health and clinical researchers and professionals have greatly improved their understanding of how to prevent type 2 diabetes, manage type 1 or type 2 diabetes effectively to reduce complications, and address disparities related to the disease. Research has identified effective, scalable interventions to address modifiable risk factors such as poor diet, obesity, and physical inactivity, that can prevent or delay type 2 diabetes (3–5) as well as interventions to teach people with diabetes how to manage their condition through lifestyle modification, medication adherence, and glucose monitoring (5). Researchers have also begun to shed light on the underlying drivers of disparities in diabetes prevalence and complications observed across socioeconomic, geographic, and racial and ethnic subgroups. Specifically, the past 50 years have seen the creation of the National Diabetes Prevention Program (National DPP) to prevent or delay the onset of type 2 diabetes among those identified at high risk (6,7), the development of effective diabetes self-management education and support (DSMES) services to reduce the risk of complications among people with diabetes (8), and recognition of the critical role that social determinants of health (SDOH) play in disparities in the risk of type 2 diabetes and its complications (9–12).

In the late 1970s, the clinical community established diagnostic criteria to identify people with early indications of glucose dysregulation or prediabetes (13). People with prediabetes have blood glucose levels higher than normal but not yet high enough to be considered diabetes (14). Currently, 98 million adults in the US have prediabetes, putting them at high risk of developing type 2 diabetes and forming a critical population for focused prevention efforts (2). In 1996, the National Institutes of Health commenced the Diabetes Prevention Program (DPP) study, a multicenter randomized clinical trial that tested the efficacy of a structured lifestyle intervention, which constituted 1 of the 3 arms of the study. The findings from the DPP trial, published in 2002, indicated a 58% reduction in the risk of developing type 2 diabetes among adults with prediabetes who engaged in the lifestyle intervention (15).

## National DPP Lifestyle Change Program

To increase implementation of type 2 diabetes prevention activities, Congress authorized the Centers for Disease Control and Prevention (CDC) to establish and manage the National DPP in 2010 (6). This partnership of public and private organizations is building a nationwide delivery system for a yearlong lifestyle change program (LCP) to help adults at high risk make modest behavior changes to prevent or delay the onset of type 2 diabetes (16). For more than 10 years, the National DPP LCP has been implemented in various settings, including workplaces (7,17). Workplaces play a crucial role in participant referral and identification, and CDC encourages employers to support their staff in taking preventive measures against type 2 diabetes and cardiometabolic diseases (18). Tsai and colleagues explored obstacles and facilitators to participant engagement in employer-sponsored clinic-based LCPs, suggesting that engagement in a workplace LCP can be supported by addressing specific workplace challenges and gaining buy-in from employers (19). Incorporating virtual approaches for delivering the National DPP in hybrid work settings promises to be an effective strategy to reduce barriers to referrals from providers (19).

Despite widespread implementation, significant challenges exist in recruiting, enrolling, and retaining participants from priority populations into the National DPP LCP (20,21,22). In this collection, authors examine National DPP LCP participation drivers, explore participant readiness to enroll, examine the use of technology to increase engagement, discuss the role of the workplace in program delivery, and provide program tailoring and adaptation recommendations to increase relevance and reach to particular racial and ethnic communities.

Saiki and colleagues (17) and Hulbert and colleagues (23) highlighted the approaches to recruit and engage racial and ethnic communities in DPP LCP programs. Saiki and colleagues identified barriers and facilitators to program recruitment and completion among native Hawaiian and Filipino populations residing in rural Hawai‘i. These barriers and facilitators suggest that programs should use trusted community members to motivate participants to enroll and that social support from lifestyle coaches and enrolled family and friends were motivators for program completion (17). Hulbert and colleagues examined the interests and barriers and facilitators for program participation and healthy behaviors in a group of non-Hispanic Black, Hispanic, Asian, American Indian or Alaska Native, and Native Hawaiian or Pacific Islander men, suggesting that program incentives, male-specific topics, and the involvement of family members may be motivators for participation (23). Likewise, Johnson and colleagues showed how technology, behavior change theories, and community-based participatory design may be promising strategies for increasing engagement in the National DPP LCP (24). These authors employ systematic research and program evaluation methods to test and refine the use of current evidence and other public health strategies. The results offer insights into the factors that influence engagement in the LCP, including the importance of tailoring programs to align with participants’ interests and preferences. Additionally, the results underscore how understanding the preferences of people at risk for type 2 diabetes can enhance participation in health programs by selecting the most effective delivery methods and locations to encourage greater involvement and improve overall outcomes.

## Addressing Diabetes Complications

People living with diabetes face an increased risk of serious complications, especially cardiovascular disease, kidney disease, eye disease, and lower limb amputations that result in substantial illness and death (25). For example, a 50-year-old adult recently diagnosed with type 2 diabetes currently has a life expectancy 6 years shorter than someone without diabetes (26). However, the reduced life expectancy associated with diabetes can be alleviated by effectively achieving treatment objectives related to glucose management, blood pressure control, and cholesterol levels to prevent complications (27). A fundamental strategy for accomplishing these treatment objectives is DSMES, which empowers individuals to effectively manage their diabetes (8). DSMES participation can improve glycemic control, management of blood pressure and cholesterol, medication adherence, nutrition, physical activity, and self-confidence to successfully manage diabetes, ultimately leading to a reduction in diabetes-related complications and decreased health care costs (28).

## Diabetes Self-Management Programs

The American Diabetes Association (ADA) and the Association of Diabetes Care & Education Specialists (ADCES) support DSMES through program accreditation and recognition and accreditation of diabetes care and education specialists (8). CDC provides funding to state and local health departments and other organizations to increase access to and participation in DSMES services (8). As of 2020, recognized or accredited DSMES programs were offered in all 50 states, including 56% of all US counties (8), and nearly 1 million people diagnosed with diabetes accessed these DSMES services (5). Despite this number, less than 10% of those newly diagnosed with diabetes participate in DSMES within the first year of diagnosis (8). Thus, finding ways to expand access to and participation in DSMES is a key approach to preventing complications among people with diabetes. Hulbert and colleagues’ work (23) regarding motivators for program participation provided insights that are useful for both National DPP and DSMES services. Simultaneously, Bing and colleagues described an approach to expanding DSMES access and enrollment by evaluating the programmatic work of state health departments, shedding light on how engaging the pharmacy sector, using an umbrella organization approach, and implementing continuous quality improvement efforts may help improve referral and enrollment in DSMES programs (29).

The burden of managing type 2 diabetes every day is substantial and can be overwhelming, affecting both mental health and the self-efficacy required for successfully preventing complications (30). This mental health impact is called diabetes distress (31). Alexander and colleagues investigated the prevalence and determinants of diabetes distress among US adults and recommended strategies that, if incorporated into interventions, could improve diabetes management (32). This study estimated the national prevalence of diabetes distress for the first time, finding that 1 in 4 adults with diabetes in the US experiences moderate or severe diabetes distress.

## Other Factors in Diabetes Prevalence Disparities

Another key development in type 2 diabetes prevention and diabetes management has been the acknowledgment of the role of upstream social and environmental factors, such as employment and financial security, education, safe and stable housing, access to nutritious food, dependable transportation, and other stressors, on type 2 diabetes prevalence disparities (10,11,33). A clear example of how SDOH can impact diabetes risk and risk factors can be seen in the rural US (34). Rural residents often struggle to access health care; the prevalence of healthy behaviors is lower and the prevalence of chronic disease is higher compared with those in urban areas (35). Khavjou and colleagues analyzed rural–urban disparities in diabetes prevalence across states among US adults (36), and Onufrak and colleagues investigated diabetes prevalence in relation to county metropolitan status and region (37). While their findings correspond with known rural–urban disparities in diabetes deaths, hospitalizations, and incidence, the authors also examine the underlying SDOH factors that contribute to observed disparities and provide a more detailed picture of how rural disparities differ across the US. Both studies suggest that rural–urban disparities in diabetes prevalence are not homogeneous across the US and suggest that such disparities are at least partially explained by socioeconomic factors. Disparities include not only differences in prevalence and risk but also in complications for those who already have diagnosed diabetes. Zhou and colleagues studied cardiovascular disease prevalence among Medicare beneficiaries with diabetes, highlighting differences by race and ethnicity, socioeconomic status, and urbanicity (38). They found that cardiovascular disease prevalence varied by race and ethnicity and that a low income-to-poverty ratio and food insecurity were positively associated with myocardial infarction, stroke, and heart failure. These findings corroborate with existing literature on income and education disparities in diabetes in the US (39,40). Saelee and colleagues examined the link between household energy insecurity and diabetes prevalence (41), shedding light on a novel SDOH that may affect illness and death among persons with diabetes (42,43). They report that states with higher prevalence of diabetes also have greater prevalence of energy insecurity, a condition which may complicate diabetes management during times of severe weather.

While evidence-based programs such as the National DPP have demonstrated effectiveness (44), challenges related to cost, accessibility, and long-term adherence remain significant barriers to widespread implementation. Telehealth and telemedicine are approaches to addressing these issues among rural populations and others facing barriers to health care access because of distance, transportation, or difficulty taking time off from work (45). During the COVID-19 pandemic, telemedicine use surged, but data on its usage among US adults with prediabetes or diabetes are limited. Zaganjor and co-authors report variations in telemedicine use based on region, urban or rural status, insurance, and education, identifying specific populations with prediabetes or diabetes that may benefit from improvements in telemedicine access (46).

CDC and its partners are dedicated to addressing factors that contribute to the onset of type 2 diabetes and inadequate management of diabetes. In the commentary “Breaking Barriers: CDC and American Diabetes Association Unite to Combat Diabetes,” authors Holliday and Gabbay detail the collaboration between CDC and ADA, along with other federal agencies, state and local health departments, health care providers, and community organizations, to combat the impact of diabetes on the nation (47). The authors specifically highlight the upstream, midstream, and downstream strategies that can be employed to improve the prevention and management of diabetes in the US.

## Conclusion and Future Directions

The US diabetes epidemic is influenced by a myriad of complex factors, suggesting innovative methods may be required to stem the tide of both diabetes and its complications and comorbidities. The articles in this collection describe and consolidate research and evaluation related to identifying barriers to the prevention and management of diabetes, and effectively implementing and evaluating evidence-based approaches aimed at fighting this pervasive disease. They illuminate the challenges faced by priority populations in their everyday environments and showcase innovative approaches in public health practice, such as tracking national initiatives and embracing new technologies. This collection highlights opportunities for further research, applied public health research, and prioritization of the use of findings from program and implementation evaluation to further improve program development. Continued coordinated efforts among multilevel partnerships across all sectors, along with evaluating and implementing emerging and promising practices as they develop, will allow us to address diabetes effectively. Future work may also prioritize interventions that improve access to care for all populations. Further, incorporating behavioral interventions such as stress management, psychoeducation, and family support into diabetes care can improve patient well-being and adherence to treatment (32). Addressing these challenges may require a comprehensive approach, including tailored interventions and innovative health care delivery models such as telemedicine and community-based programs. In sum, the findings featured in this collection can, in various ways, help guide specific, focused interventions to reduce disparities in diabetes prevalence and complications.
